# Effects of Educational Music Therapy on State Hope for Recovery in Acute Care Mental Health Inpatients: A Cluster-Randomized Effectiveness Study

**DOI:** 10.3389/fpsyg.2016.01569

**Published:** 2016-10-07

**Authors:** Michael J. Silverman

**Affiliations:** School of Music, University of MinnesotaMinneapolis, MN, USA

**Keywords:** hope, educational, mental health, music therapy, acute psychiatric patients, recovery, state hope

## Abstract

**Background:** There has been an increasing emphasis on recovery as the expectation for people with mental health disorders.

**Purpose:** The purpose of this effectiveness study is to determine if group-based educational music therapy can immediately impact state hope for recovery in acute care mental health patients. Research questions included: will acute care mental health inpatients who participate in a single music therapy session have higher agency and pathway aspects of state hope for recovery than patients in a control condition? Will there be differences in state hope for recovery as a result of hope-oriented songwriting or lyric analysis interventions?

**Method:** Participants (*N* = 169) were cluster randomized to one of three single-session conditions: lyric analysis, songwriting, or wait-list control.

**Results:** There was no significant between-group difference. However, both music therapy conditions tended to have slightly higher mean pathway, agency, and total state hope scores than the control condition even within the temporal parameters of a single music therapy session. There was no between-group difference in the songwriting and lyric analysis interventions.

**Conclusion:** Although not significant, results support that educational music therapy may impact state hope for recovery within the temporal parameters of a single session. The specific type of educational music therapy intervention did not affect results. Implications for practice, limitations, and suggestions for future research are provided.

## Introduction

For people with mental health disorders, there has been a rising commitment to and emphasis on recovery as the expectation (Repper and Perkins, 2003; Davidson and Roe, 2007). In the unique contextual parameters of mental illness, recovery can be defined as a process of developing new self-directed meaning and purpose (Anthony, 1993; Lunt, 2000). In this model, recovering a meaningful, purposeful, and valued life is more important than the removal of mental health problems. Thus, health care professionals can collaboratively work to support consumers to develop and enhance their quality of life by facilitating hope, opportunity, and control (Repper and Perkins, 2003).

Many definitions of recovery include hope as a critical component. For example, by studying operational definitions of recovery derived from clinical observations and interactions between professionals and consumers, Noordsy et al. (2002, p. 318) conceptualized that recovery consisted of three components: “(a) hope; (b); taking personal responsibility for illness management and wellness; and (c) ‘getting on with life’ beyond illness”. Jacobson and Greenley (2001) noted recovery from mental illness can refer to both internal and external conditions. Internal conditions include hope, healing, empowerment, and connection while external conditions include implementation of the principle of human rights, a positive culture of healing, and recovery oriented services (Jacobson and Greenley, 2001). Scholars have also recognized hope as a central construct in the recovery process: “Hope is recognized as one of the most critical determinants of recovery from mental illness” (Noh et al., 2008, p. 69). Other authors have also noted that hope represented an integral component of the recovery process (Anonymous, 1989; Russinova, 1999; Noordsy et al., 2002). Corrigan (2003, p. 349) stressed the importance of hope for people with mental health disorders: “…that most of the experiences that define essential humankind are still attainable”. Russinova (1999) noted that hope concomitantly functions as a source and an outcome of the recovery process. Thus, in these aforementioned definitions of recovery, hope consistently represents an integral and defining component of the model and process.

Hope can be conceptualized as the achievement of goals that have relevance and significance for the person (Gudjonsson et al., 2011). Scioli et al. (1997) noted that hope is an emotion that can subsequently affect motivation and therefore influence beliefs and behaviors. Moreover, hope is associated with psychosocial benefits (Snyder et al., 2011) and appears to be an important component of therapeutic change (Yalom, 1995; Snyder and Lopez, 2009). In psychotherapeutic encounters, therapists and patients have regularly recognized hope as a key factor (Sayin et al., 2008). Weis and Speridakos (2011) conducted a meta-analytic review on hope in clinical and community settings and found that hope enhancement strategies were associated with significant increases in self-reported hopefulness. The researchers also found single-session interventions to have larger effect sizes (*d* = 0.40) than interventions administered over multiple sessions (*d* = 0.19). Thus, it seems that hope is malleable and can be experimentally manipulated, particularly in short-term and acute care settings. These data provide implications for hope as a target construct for clinicians to address specifically in acute care mental health settings.

Snyder et al. (1996), recognized as scholarly authorities concerning hope, noted goal directed cognitions are important qualities in the attainment of constructive outcomes. Therefore, hope can be conceptualized as a positive motivational cognitive set that is based on a commonly derived sense of both agencies and pathways (Snyder et al., 1991a,b). Agency refers to the person’s perceived capacity for initiating and maintaining the actions necessary to accomplish a goal. Weis and Speridakos (2011) noted agency self-statements can include “I can accomplish this task” and “I will not be stopped.” Pathways refer to the person’s perceived ability to generate plausible routes to goals. Weis and Speridakos (2011) noted pathway self-statements can include “I can find a way to get this done” and suggested that both agency and pathway components of hope can be validly measured. It should be noted, however, that both agency and pathway cognitions are necessary for higher overall hope.

Specific to psychotherapeutic encounters, Snyder et al. (2000, p. 257–258) noted that hope represented a common factor: “Psychotherapies ‘work’ precisely because they enable people to identify goals that represent solutions to their problems, they specify particular routes for reaching those goals (pathways thinking), and they motivate clients to use those routes so as to implement change (agency thinking)”. Thus, a therapist’s responsibility can be conceptualized as to increase hope by helping clients set objective and clear goals and by intensifying agency and pathway cognitions (Weis and Speridakos, 2011). Yalom (1995) also noted hope was a key factor in group-based psychotherapeutic encounters.

Concerning mental health recovery, state indices of hope – including agency and pathway cognitions – can be essential as hope and optimism have contributed to understanding how people cope with stressors. State hope can provide a “snapshot” of a person’s goal-directed cognitive processes (Snyder et al., 1996, p. 321). Theoretically, people with higher state (i.e., in the present moment) hope participate in more positive events and have lower depression levels (Snyder et al., 1997) and have higher academic achievement (Snyder et al., 2002b). People with lower hope tend to have negative outcomes and reductions in wellbeing (Diener, 1984). From a recovery perspective, hope is consequential as people with higher state hope will likely be engaged and motivated to participate in their psychotherapeutic and psychoeducational treatments.

Numerous researchers have found hope to be related to functional outcomes in patients diagnosed with schizophrenia (Regenold et al., 1999; Hoffmann et al., 2000; Lysaker et al., 2001; Roe, 2003). Other researchers have suggested that hope mediates the relationship between insight and quality of life in people with schizophrenia and that hope is negatively correlated with distress, anxiety, and depression while it is positively correlated with subjective health, self-efficacy, and personal resilience in people with mental health disorders (Schrank et al., 2008). Thus, increasing hope may directly and positively lead to increases in quality of life and the patients’ self-awareness concerning their mental illnesses (Hasson-Ohayon et al., 2009).

Unfortunately, many people with mental health disorders feel hopeless (Babow and Rowe, 1993; Harding and Zahnister, 1994; Kirkpatrick et al., 1995). Specifically, Deegan (1988) noted that people with mental illnesses often describe periods of hopelessness prior to their recovery process. In order to modify cognitive, behavioral, and affective components, hope for change is a necessary component of motivation. Motivation, in turn, is a necessary component for taking action in attempts to meet goals (Miller and Rollnick, 2002). Attitudinal components of hope include cultivating optimism, reordering priorities, recognizing and accepting that a problem exists, commitment to change, focusing on strengths rather than weaknesses, and forward thinking (Jacobson and Greenley, 2001).

A person’s degree of hope may also influence coping mechanisms, which constitute an important aspect of illness management and recovery. Lysaker et al. (2005) described a relationship between hope and coping mechanisms: people cope better and by utilizing more effective mechanisms when they are hopeful. These authors predicted that patients with schizophrenia who were more hopeful would prefer active problem solving and found that patients who had higher levels of hope had greater preference for positive coping skills such as action and lesser preferences for negative coping skills such as resigning and ignoring. Hope also constitutes an issue for people coping with serious mental illness while they are in outpatient care. In a qualitative investigation, outpatients with serious mental illnesses emphasized the importance of hope for recovery as well as practitioner optimism in treatment (Lester et al., 2005).

While music therapy research concerning the recovery model exists, this literature consists mostly of qualitative, review, and position papers (Chhina, 2004; Grocke et al., 2008; Kooij, 2009; McCaffrey and Edwards, 2016; McCaffrey et al., 2011; Solli et al., 2013; Silverman, 2015). To date, there is no quantitative music therapy literature specifically targeting hope. However, in a qualitative investigation related to recovery, Ansdell and Meehan (2010) found a key benefit of music therapy for mental health patients is its ability to mobilize music’s hope. Additionally, no randomized music therapy studies examining the recovery model – or specific components of recovery, including hope – exist. Since hope can influence coping and represents a vital component of recovery, studies concerning state hope in people with mental illnesses are warranted (Noh et al., 2008). As hope is considered a malleable construct (Snyder, 1994; Snyder et al., 2002a; Weis and Speridakos, 2011), experimentally manipulating state indices of hope within a psychotherapeutic treatment intervention could lead to enhanced coping, which may facilitate improved functional and clinical outcomes and augmented illness management skills. Therefore, the purpose of this cluster-randomized effectiveness study is to determine if group-based educational music therapy can immediately impact state hope in acute care mental health inpatients. The primary research question was as follows: will acute care mental health inpatients who participate in a single educational music therapy session have higher agency and pathway aspects of state hope than participants in a control condition? A secondary question concerned potential differences between the effectiveness of specific music therapy interventions: will there be differences in state hope as a result of educational songwriting or lyric analysis interventions?

## Materials and Methods

### Participants

Research participants (*N* = 169) were inpatients on an acute care mental health unit. Congruent with existing data indicating the length of a patient’s stay in a mental health facility has reduced to 7.2 days per admission (Centers for Disease Control and Prevention, 2015), patients typically remained on inpatient status for 3–7 days and had varied diagnoses (see **Table 1**), including bipolar disorder, major depressive disorder, schizoaffective disorder, and schizophrenia. Although patients typically had multiple diagnoses, the researcher only used the primary diagnosis in an attempt to simplify this effectiveness study. The unit was part of a large university hospital in the Midwestern region of the United States. Patients volunteered to take part in the study, were not paid, and had the option to receive treatment without being a research participant. Eligibility criteria included all patients admitted to the inpatient acute care unit attending their first music therapy session. Exclusion criteria included not being able to read English and attending multiple sessions with the researcher. Criteria were purposely inclusive in order to provide treatment to as many patients as possible and accurately represent contemporary acute mental health care in this effectiveness study.

**Table 1:** Frequencies of gender, race/ethnicity, and primary diagnosis.

		Lyric analysis	Songwriting	Wait-list control
Gender				
	Female	37	29	25
	Male	28	25	25
Race/ethnicity				
	African American	9	7	3
	Caucasian	48	40	39
	Hispanic	3	1	0
	Native American	2	4	5
	Other	2	1	2
	No response	1	1	1
Primary diagnosis				
	Anxiety disorder	4	0	1
	Bipolar disorder	15	20	13
	Major depressive disorder	30	20	21
	Post traumatic stress disorder	5	0	2
	Schizoaffective disorder	2	5	5
	Schizophrenia	0	1	0
	Psychosis	1	1	0
	No response	8	9	8

*All p values are greater than 0.05*.

Patients on the unit were scheduled to attend a variety of daily group-based psychosocial and psychoeducational programming sessions; these included community meetings, general health, occupational therapy, process group, medication education, spirituality, topics group, reflection/journal sessions, and relaxation group. None of the available sessions specifically addressed hope for recovery. The researcher provided one of the three group-based treatment conditions each week, for a total of 42 sessions.

### Design

Due to the unique contextual and temporal parameters of acute mental health care, this study was a single-session, cluster-randomized three-group design. A single-session design was selected because it is more congruent with the rapid ebb and flow of patients characteristic of the acute care mental health setting than other research designs. Questionnaires were only utilized once per session in an attempt to minimize measurement fatigue within the temporal parameters of a single treatment session. The investigator, a board certified music therapist with over 13 years of clinical mental health experience at the onset of the study, conducted all sessions.

Congruent with terminology from Silverman (2015), the investigator adopted the term *educational* from Murray-Swank and Dixon (2005), who referred to brief family based psychoeducational models as family *educational* programs (Murray-Swank and Dixon, 2005). As the current study was conducted on an acute care mental health unit, the investigator used the term *educational* instead of *psychoeducational* to differentiate the single-session treatment from typically longer-term and higher dose psychoeducational treatments. The goal of the educational conditions was based from the illness management and recovery literature and involved increasing patients’ hope and motivation for recovery. This approach also shares the collaborative and empowering aspects of resource-oriented music therapy (Rolvsjord, 2010), but the acute care nature of the crisis stabilization unit – wherein single session therapy was the norm – necessitated a more time limited and direct approach.

Participants who attended study sessions were cluster randomized into conditions by session, with a total of 42 sessions delivered during the study period. Cluster randomization methods have become popular and involve randomizing social units rather than individuals (Laupaiboon, 2003; Guittet et al., 2006). In the current study, the investigator randomized the numbers 1–42 into three groups and each group was assigned to a condition. The only differences between clusters were the independent variables delivered: participants were on the same acute care inpatient mental health unit and all other between-group aspects were similar. Patients could voluntarily become research participants in the first session they attended. In an attempt to be as inclusive as possible, participants were allowed to attend multiple sessions but data were only collected after completion of a participant’s first session. Each morning before the session began, the investigator announced “music group” would be starting and encouraged all patients on the unit to attend. Thus, patients had a choice to attend sessions but did not choose what condition would take place on each day. Regardless of treatment condition, staff members on the unit also encouraged patients to attend sessions. The investigator conducted the study Tuesday mornings from June 2013 to May 2014.

All sessions were group-based and approximately 45-min and were scripted. Group-based music therapy is common in the United States (Silverman, 2007) and within acute mental health care (Thomas, 2007). Regardless of condition, the investigator described and obtained informed consent from each group of patients at the immediate onset of each session. During this process, the investigator explained the study including potential risks and benefits of participation, that study participation was voluntary and participants could withdraw from the study at any time without penalty, and what participation would involve. Given the clinical population and setting, the Institutional Review Board granted a waiver of signatures on informed consent forms to augment confidentiality. Although all consent procedures were group-based, potential participants were able to individually ask the investigator questions. In an attempt to be as inclusive as possible within the contextual parameters of an acute mental health unit, patients were also permitted to attend sessions without being research participants. Due to the exploratory nature of the study, ethical implications, and issues of confidentiality at the facility, no fidelity measures were taken. The University of Minnesota Institutional Review Board approved this study.

### Instrument

Based on the premise that a person’s cognitions about goal-directed activities constitutes an important aspect of attaining positive outcomes, the State Hope Scale (SHS; Snyder et al., 1996) is a six-item instrument composed of agency (three items) and pathway (three items) subscales as well as a total State Hope score (six items). Snyder et al. (1991a) noted that agency and pathway components of hope parallel the self-efficacy and outcome expectancies of social cognitive theory. As persons can believe in their abilities to act without being aware of how to research a goal – or vice versa – the agency and pathway subscales are considered additive and reciprocal but not synonymous (Lyndall, 2002).

The SHS can be used as an instrument to further understand meditational processes between antecedent and consequent events and the creators noted the SHS “appears to meet the psychometric standards for self-report scales” (Snyder et al., 1996, p. 334). Items are scored on an eight-point Likert-Type Scale with one representing “Definitely False” and eight representing “Definitely True.” Subscale scores can range from three to 24 and the total hope scores can range from six to 48, with higher scores representing greater hope levels. In the development and testing of the SHS, Snyder et al. (1996, p. 334) found internal reliability ranged from 0.82 to 0.95 with a median alpha of 0.93 and wrote, “the scale exhibits discriminant validity in that it cannot be explained in terms of other state self-report indices related to social desirability, self-esteem, positive and negative affectivity, and academic performance” (Snyder et al., 1996, p. 334). In a comprehensive review paper concerning hope in psychiatry, Schrank et al. (2008, p. 430) noted that the State Hope Scale was frequently used in mental health research, was ideal for routine clinical use, and that it exhibited “robust psychometric properties”.

### Intervention Design

In a qualitative study, Darlington and Bland (1999) interviewed experienced mental health workers and consumers concerning fostering and maintaining a sense of hope. The researchers identified similar themes in both groups. Themes from the mental health workers included: working within the client’s frame of reference, focusing on the client’s strengths, making links to past gains, being human, and having hope that change is possible. The themes that emerged from consumers included: using knowledge of mental illness to help clients, helping clients to understand their illness, understanding the importance of achievement, being genuine, being realistic about what can be achieved, and holding on to hope when the client has none. These identified themes guided the development of the music therapy interventions designed to target state hope.

In a paper utilizing grounded theory to describe how community mental health nurses promote hope in young adult clients diagnosed with schizophrenia, the author noted that the main strategies participants employed to uncover hope were to enhance motivation and develop pathways to wellness (McCann, 2002, p. 95:

Enhancing motivation takes place within the context of a supportive relationship and attempts to uncover clients’ philosophy of life-what provides meaning or hope in their lives. Motivation cannot be imposed on clients; it needs to be uncovered, enabled, and encouraged. Associated with enhancing motivation is developing pathways to wellness. The process involves working cooperatively with clients, enabling them to set realistic goals, and together, planning strategies to enable fulfillment of their goals.

Thus, the current investigator utilized these theories and focused on enhancing motivation by identifying patient-identified goals and motivators and developing pathways to recovery by identifying how participants could reach their goals within the music therapy interventions. Moreover, the two educational music therapy interventions – songwriting and lyric analysis – were also based from Snyder’s theory reflecting hope as a malleable cognitive construct that insinuates people’s motivation and capability to strive toward personally pertinent ambitions (Snyder, 1994; Snyder et al., 2002a). In this construct, the therapist helps the client to identify and select relevant and meaningful goals and then facilitates the generation of multiple pathways toward the accomplishment of the goals.

Songwriting and lyric analysis interventions are frequently used with mental health patients (Silverman, 2007). As this was a group-based single-session effectiveness study with acute care mental health inpatients, the researcher wanted to be consistent with contemporary clinical practice, so songwriting and lyric analysis were appropriate given the contextual and temporal parameters. Interventions were derived and constructed from music therapy research (Silverman, 2013a,b, 2014, 2015) and, more specifically, lyric analysis (Silverman, 2009, 2014, 2015) and songwriting (Silverman, 2013a,b, 2014, 2015; Baker, 2015) literature.

### Conditions

#### Lyric Analysis

In an introductory exercise, the investigator first asked participants to state their names and something about themselves (such as favorite food, least favorite vegetable, or favorite sport) within a 12-bar blues progression in the Key of E played on an acoustic guitar (Yamaha FG720S) to expediently establish rapport. The investigator then distributed small pieces of paper and pens to all patients and instructed them to write the title of a song that made them hopeful and motivated them. The investigator then collected these papers, read each song title, and the patients’ task was to identify which patient wrote each song. After each patient’s song was identified, the investigator asked the patient for what she or he was hopeful and what she or he could do to facilitate attaining self-directed goals. The investigator then distributed lyrics sheets for the song “Times like these” and played and sang the song, asking patients to circle any lyrics that “jumped out” at them or to which they could relate. After playing the song, the investigator facilitated a dialog concerning patients’ identified lyrics and how they related to hope for recovery. At the conclusion of the session, the investigator verbally processed the session, thanked patients for participation, asked for questions and comments about the session, and distributed posttests to patients who volunteered to complete them.

#### Songwriting

In an introductory exercise, the investigator first asked participants to state their names and something about themselves (such as favorite food, least favorite vegetable, or favorite sport) within a 12-bar blues progression in the Key of E played on an acoustic guitar to expediently establish rapport. The investigator then informed patients they would be writing a song about hope for recovery but they would begin by brainstorming ideas of why patients wanted recovery for the first verse. The investigator wrote down patients’ ideas and suggestions in a type of “lyric bank” on a dry erase board. Common ideas concerned family, friends, health, vocational aspirations, and happiness. The investigator then facilitated group songwriting utilizing patients’ suggestions. Thus, the first verse of the blues song concerned why patients wanted recovery and therefore focused on motivators (i.e., pathways). The investigator used a similar procedure for the second verse but focused on how patients would recover (i.e., agency). Common ideas concerned taking medications as prescribed, adhering to therapy as an outpatient, monitoring the illness, and having a positive attitude. The songs were 12 bar blues songs in the key of E and, due to time constraints of a single therapy session on the acute care unit, patients only had to write lyrics. Patients thus wrote a two-verse song, with the first verse concerning *why* they wanted to recover (pathway), and the second verse concerning *how* they would recover (agency). The investigator sung the song during the songwriting process and patients sometimes spontaneously sang along. At the conclusion of the session, the investigator verbally processed the session, thanked patients for participation, asked for questions and comments about the session, and distributed posttests to patients who volunteered to complete them. The investigator made copies of the song lyrics for patients to keep.

#### Wait-list Control

At the onset of the session, the investigator distributed pretests to patients who volunteered to complete them. The investigator asked participants to state their names and something about themselves (such as favorite food, least favorite vegetable, or favorite sport) to expediently establish rapport. The investigator then led the group in playing rock and roll bingo. Each patient had a separate card with 25 song titles on it. After patients heard a song played from a portable stereo, they placed a small piece of paper on the corresponding song title on their bingo card. Although patients may have learned musical information concerning artists and songs used in the rock and roll bingo game, the investigator designed the wait-list control condition to be non-educational concerning increasing hope for recovery.

### Participant Enrollment

Participants were enrolled in the study from July 2013 to May 2014 resulting in a sample of 169. This sample size enabled the researcher to detect a moderate effect size (0.25) when α = 0.05 for a power of 0.95 using an ANOVA with fixed effects with three independent treatment groups using a linear mixed model (Kotrlik et al., 2011).

### Analyses

Five separate analyses of variance (ANOVA) were conducted to determine if there were differences between the three treatment groups in (a) ages; (b) the total number of times patients had been admitted to a mental health facility; (c) the number of days they had been on inpatient status; (d) the number of patients taking part in each session who volunteered to be research participants; and (e) the total number of patients in each session. Chi-square tests were conducted to determine if there were between-group differences in the four treatment groups in categories concerning (a) gender, (b) race/ethnicity, and (c) primary mental health diagnosis.

The investigator fit a linear mixed model with group as a fixed effect and cluster as a random effect using the univariate function in SPSS version 19.0. The investigator checked to ensure that assumptions needed for the statistical tests were met. Levene’s Tests of Equality of Error Variances were not significant (agency *p* = 0.505; pathway *p* = 0.606; total hope *p* = 0.599). The investigator reported the overall *F* test for group differences.

## Results

**FIGURE 1:**
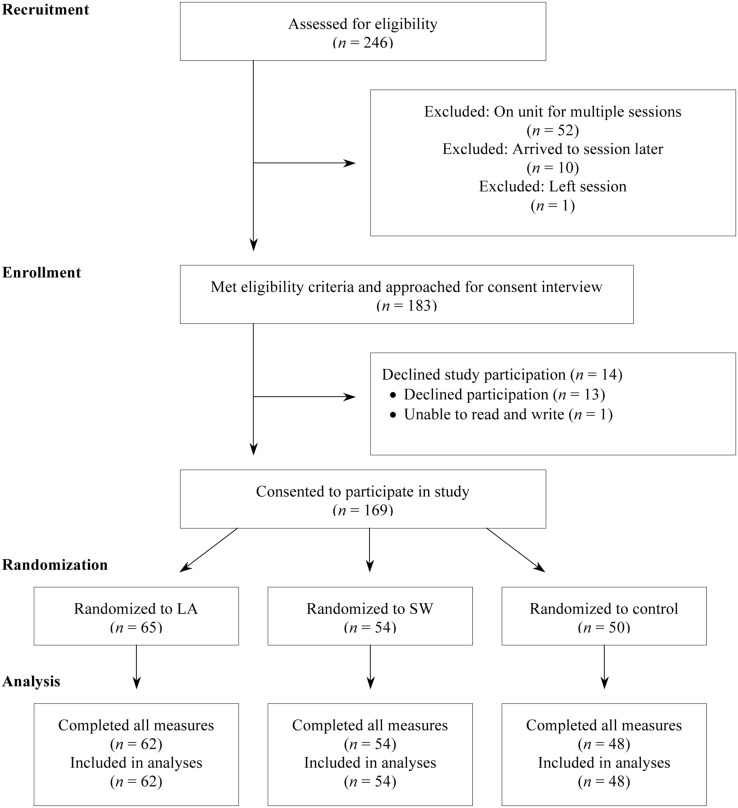
**Participant Flow**.

**Figure 1** depicts participant flow through phases of the study.

No statistically significant between-group difference was found for demographic measures, all *p* > 0.05. No statistically significant between-group difference was found in demographic frequencies, all *p* > 0.05. Participant demographic information is depicted in **Tables 1** and **2**.

**Table 2:** Descriptive statistics – independent variables.

Variable	Lyric analysis			Songwriting			Wait-list control		
	*M*	*SD*	*n*	*M*	*SD*	*n*	*M*	*SD*	*n*
Age	37.89	12.80	64	37.33	12.34	54	35.76	11.97	49
Previous mental health admissions	3.60	5.39	65	4.31	6.91	54	4.60	4.97	50
Days in hospital	5.54	5.76	65	5.51	4.16	54	5.87	6.92	50
Research participants per session	4.64	1.39	14	3.93	1.59	14	3.57	1.83	14
Total patients per session	6.14	1.70	14	6.21	2.42	14	6.21	2.43	14

*All p values are greater than 0.05*.

There was no significant between-group difference for agency [*F*(2,161) = 2.708, *p* = 0.070, $\eta_{p}^{2}$ = 0.033], pathway [*F*(2,161) = 2.767, *p* = 0.066, $\eta_{p}^{2}$ = 0.033], or total state hope [*F*(2,161) = 2.940, *p* = 0.056, $\eta_{p}^{2}$ = 0.035]. Thus, there was no difference between music therapy conditions and the control (research question 1) and no difference between the two educational music therapy conditions (research question 2). Although not significant, descriptive data indicated that both music therapy conditions tended to have slightly higher mean agency, pathway, and total hope scores than the control condition (research question 1). While both music therapy conditions had higher means than the control condition, between music therapy intervention differences were minimal (research question 2). State hope descriptive statistics by treatment group are depicted in **Table 3**.

**Table 3:** Descriptive statistics – state hope.

	Lyric analysis			Songwriting			Wait-list control		
Variable	*M*	*SD*	*n*	*M*	*SD*	*n*	*M*	*SD*	*n*
Agency	18.48	4.82	62	18.50	4.23	54	16.58	5.29	48
Pathway	17.85	4.77	62	17.69	4.08	54	15.93	4.94	48
Total state hope	36.34	9.27	62	36.20	9.03	54	32.51	9.80	48

*All p values are greater than 0.05*.

## Discussion

The purpose of this cluster-randomized effectiveness study was to determine if group-based educational music therapy could immediately impact state hope for recovery in acute mental health patients. Results indicated that there was no difference between music therapy and the control conditions and no difference between the two music therapy conditions. However, when considered within the unique contextual and temporal parameters of group-based acute care single-session music therapy, results may have clinical implications that educational music therapy can immediately impact state hope. Although not significant, descriptive data indicated that both music therapy conditions tended to have slightly higher mean agency, pathway, and total hope scores than the control condition. While both music therapy conditions had higher means than the control condition, between music therapy intervention differences were minimal. Additionally, Weis and Speridakos (2011) conducted a meta-analytic review on hope and found larger effect sizes when interventions were administered by researchers in the context of laboratory-based research than hospital settings. As the current effectiveness study was conducted on an acute care inpatient mental health unit, potential between-group differences may have been minimized due to the setting. While differences were not significant and generalizations are inappropriate, there tended to be slight differences in descriptive data even within a single treatment dose common in acute care inpatient settings.

Although the investigator purposely did not target traditional symptoms in the current study, results may also have implications concerning symptoms of mental illness. For example, Lysaker et al. (2005) found that higher levels of hope were associated with lower positive symptom levels. Additionally, Hasson-Ohayon et al. (2009) found a direct positive relation between hope and a number of quality of life components.

Anecdotally, the educational music therapy interventions were well received by patients and the investigator attributes some of this success to the high degree of structure within the educational music therapy interventions. Visiting nursing students observing the sessions commented how patients verbally participated more during music therapy than during other group-based programming. One patient wrote a note to the CR on the back of his questionnaire without prompting: “I feel better and more hopeful after hearing you talk and sing/play for us. Thank you for coming. I hope you make it back sometime.” Another patient, who noted that creative writing functioned as a coping skill for him, extemporaneously wrote the clinician a haiku: “I feel better now, because Michael sang to me, so thanks.” During the songwriting condition, one participant spontaneously wrote a note to the researcher concerning the song the group composed: “Loved it and will cherish it forever.” This comment is particularly interesting as the songs composed during sessions were purposely educational and group-based. It seems that this participant had a strong and positive affective reaction to the group-based songwriting process and product even though the song was purposely educational and not as personalized as a song would be if written during an individual music therapy session.

Limitations of the study can initiate with the absence of follow-up measures to determine maintenance of treatment gains as patients’ hope may have dissipated immediately after the session. Due to the high degree of patient turnover and single-session design applicable on an acute care unit, the investigator did not administer measures beyond the session. Additionally, the investigator’s dual role of researcher and music therapy clinician may have led to biased participant responses and also represents a limitation. Finally, due to the nature of the acute care mental health unit, hope was measured via a cognitive construct (i.e., agency and pathway). As such, the current study cannot generalize to hopeful behaviors.

Future research – using various paradigms to more holistically understand if and how hope may be affected – is warranted to determine if educational music therapy targeting hope has other beneficial results. Additionally, although it is difficult to objectively measure the experience of songwriting from the patients’ perspectives and how the experience may relate to clinical outcome, future research is warranted to better understand songwriting processes and products. In future studies, research assistants could collect data to minimize bias. Moreover, multiple data collection sites and therapists would increase sample size and better test the protocols used. Finally, follow-up measures consisting of more traditional metrics could be conducted to determine depression, quality of life, and recidivism.

The purpose of this randomized effectiveness study was to determine if group-based music therapy can immediately impact state hope in acute mental health patients. Results indicated that there was no difference between music therapy conditions and the control condition and no difference between the two music therapy conditions. However, descriptive data indicated that both music therapy conditions tended to have slightly higher mean agency, pathway, and total hope scores than the control condition and between music therapy intervention differences were minimal. Future research concerning hope as well as other recovery-related constructs using a variety of research paradigms is warranted to better understand effects of music therapy with acute care mental health consumers.

## Author Contributions

The author confirms being the sole contributor of this work and approved it for publication.

## Conflict of Interest Statement

The author declares that the research was conducted in the absence of any commercial or financial relationships that could be construed as a potential conflict of interest.
